# Nitrosonifedipine Ameliorates the Progression of Type 2 Diabetic Nephropathy by Exerting Antioxidative Effects

**DOI:** 10.1371/journal.pone.0086335

**Published:** 2014-01-28

**Authors:** Keisuke Ishizawa, Yuki Izawa-Ishizawa, Noriko Yamano, Maki Urushihara, Takumi Sakurada, Masaki Imanishi, Shoko Fujii, Asami Nuno, Licht Miyamoto, Yoshitaka Kihira, Yasumasa Ikeda, Shoji Kagami, Hiroyuki Kobori, Koichiro Tsuchiya, Toshiaki Tamaki

**Affiliations:** 1 Department of Medical Pharmacology, Institute of Health Biosciences, The University of Tokushima Graduate School, Tokushima, Japan; 2 Department of Pharmacology, Institute of Health Biosciences, The University of Tokushima Graduate School, Tokushima, Japan; 3 Department of Pediatrics, Institute of Health Biosciences, The University of Tokushima Graduate School, Tokushima, Japan; 4 Department of Pharmacology, Faculty of Medicine, Kagawa University, Kagawa, Japan; Fondazione IRCCS Ospedale Maggiore Policlinico & Fondazione D'Amico per la Ricerca sulle Malattie Renali, Italy

## Abstract

Diabetic nephropathy (DN) is the major cause of end-stage renal failure. Oxidative stress is implicated in the pathogenesis of DN. Nitrosonifedipine (NO-NIF) is a weak calcium channel blocker that is converted from nifedipine under light exposure. Recently, we reported that NO-NIF has potential as a novel antioxidant with radical scavenging abilities and has the capacity to treat vascular dysfunction by exerting an endothelial protective effect. In the present study, we extended these findings by evaluating the efficacy of NO-NIF against DN and by clarifying the mechanisms of its antioxidative effect. In a model of type 2 DN (established in KKAy mice), NO-NIF administration reduced albuminuria and proteinuria as well as glomerular expansion without affecting glucose metabolism or systolic blood pressure. NO-NIF also suppressed renal and systemic oxidative stress and decreased the expression of intercellular adhesion molecule (ICAM)-1, a marker of endothelial cell injury, in the glomeruli of the KKAy mice. Similarly, NO-NIF reduced albuminuria, oxidative stress, and ICAM-1 expression in endothelial nitric oxide synthase (eNOS) knockout mice. Moreover, NO-NIF suppressed urinary angiotensinogen (AGT) excretion and intrarenal AGT protein expression in proximal tubular cells in the KKAy mice. On the other hand, hyperglycemia-induced mitochondrial superoxide production was not attenuated by NO-NIF in cultured endothelial cells. These findings suggest that NO-NIF prevents the progression of type 2 DN associated with endothelial dysfunction through selective antioxidative effects.

## Introduction

Diabetic nephropathy (DN) is one of the main reasons hemodialysis is required in patients with renal dysfunction and markedly compromises the quality of life [1]. DN is characterized by proteinuria and pathological changes in the kidney, such as glomerular hypertrophy, nodular lesions, and renal tubule injury. Such deleterious changes in the diabetic kidney are caused by oxidative stress in response to an excess amount of reactive oxygen species (ROS) [2], [3]. Prolonged hyperglycemia may be a major source of ROS, which is involved in the generation of superoxide in mitochondria [2], [4]. In the case of type II diabetes, inflammatory responses accompanied by insulin resistance also increase ROS generation, in part through the activation of NADPH oxidase [3], [5]. In addition, the activation of the intrarenal renin-angiotensin system (RAS) increases oxidative stress in the diabetic kidney [6]. Conversely, RAS activation is triggered by a ROS-mediated process that leads to an increase in angiotensinogen (AGT). It has also been shown that renal AGT expression and urinary AGT levels exhibit increases that are consistent with the diabetic condition [7], [8]. Thus, evidence suggests that the generation of ROS and AGT is markedly increased once the vicious cycle of hyperglycemia and inflammation increasing ROS, AGT, and angiotensin II (Ang II) to further enhance ROS and AGT is activated in the DN kidney [9].

Nitrosonifedipine [2,6-dimethyl-4-(2-nitrosophenyl)-3,5-pyridinedicarboxylic acid dimethyl ester] (NO-NIF) is a nitroso analog of nifedipine, which is an L-type Ca^2+^-channel blocker. Nifedipine in an alcohol solvent is extremely light sensitive, and can be converted to a photolytic compound, NO-NIF, under normal room light [10], [11], [12]. Although the ability of NO-NIF to block calcium channels is quite weak [12], its radical scavenging ability is more potent than that of nifedipine [13]. Therefore, we have focused on NO-NIF as a new therapeutic candidate against oxidative stress-related cardiovascular disease because of this antioxidative potential.

NO-NIF is highly reactive with lipid-derived radicals *in vitro*, and participates in radical scavenging activity at the cell membrane [13], [14], [15], [16]. We recently demonstrated that NO-NIF ameliorated the vascular remodeling induced by Ang II treatment in mice independent of its blood pressure-lowering effects [17]. In addition, we showed that NO-NIF restored acetylcholine-responsive vascular relaxation and suppressed intercellular adhesion molecule (ICAM)-1 expression in the aorta of *N* ^ω^-nitro-l-arginine methyl ester (l-NAME)-treated rats, a model of vascular endothelial dysfunction [18]. NO-NIF reduced the cytotoxicity of tumor necrosis factor (TNF)-α and also reduced the effect of cumene hydroperoxide to induce oxidative stress and disturb the integrity of the cell membrane in cultured human glomerular endothelial cells (HGECs) [16]. Thus, we postulate that NO-NIF is effective against endothelial cell (EC) injury and cardiovascular diseases because of its antioxidative capacity.

In the present study, we used animal models and *in vitro* evaluations to determine the efficacy of NO-NIF against DN and to examine the mechanisms of the NO-NIF antioxidative effect.

## Materials and Methods

### Ethics statement

These studies conformed to the Guide for the Care and Use of Laboratory Animals (NIH Publication No. 85–23, 1996). All animal procedures were performed in accordance with the guidelines of the Animal Research Committee of the University of Tokushima Graduate School, and the protocols were approved by the Tokushima University Institutional Review Board for animal protection.

### Chemicals and reagents

Nifedipine [1,4-dihydro-2,6-dimethyl-4-(2-nitrophenyl)-3,5-pyridinedicarboxylic acid dimethyl ester], hydrogen peroxide (H_2_O_2_), and 3-(4,5-dimethyl-2-thiazolyl)-2,5-diphenyl-2H-tetrazolium bromide (MTT) were purchased from Wako (Osaka, Japan). Dihydroethidium (DHE) was purchased from DOJINDO (Kumamoto, Japan). The anti-ICAM-1 antibody was purchased from Santa Cruz Biotechnology, Inc. (Santa Cruz, CA, USA). The anti-mouse/rat AGT antibody was obtained from Immuno-Biological Laboratories (Takasaki, Japan).

### Preparation of NO-NIF

NO-NIF was prepared from nifedipine as described in our previous report [15]. Briefly, 500 mL of nifedipine solution (10 mmol/L) in methanol was placed in a glass beaker and then exposed to halogen light (500 W, Kodak Ektagraphic III Projector, Kodak, Rochester, NY, U.S.A.) with constant stirring. Every 2 h, a sample was subjected to HPLC (H_2_O:MeOH, 4∶6) with UV detection. The eluent corresponding to newly observed peaks was collected and evaporated for determination of its structure by ^1^H-NMR, ^13^C-NMR, IR, and ESI-MS. After irradiation for 18 h under our experimental conditions, nifedipine was completely converted to NO-NIF, with a purity of more than 99%.

### Cell culture

The HGECs and CSC complete media were purchased from Cell Systems Corporation (Kirkland, WA, USA). Human mesangial cells (HMCs) and MsBM media were purchased from Cambrex Corporation (East Rutherford, NJ, USA). For experiments, the cells were maintained in media from passage 3 to 8 and used after 24–48 h of serum depletion. The human proximal tubular cell line HK-2 (American Type Culture Collection, Manassas, VA, USA) was maintained in RPMI 1640 medium (Nacalai Tesque, Kyoto, Japan) supplemented with 10% fetal bovine serum (Invitrogen, Eugene, OR, USA).

### Experimental animals and treatment

All animals were housed in a temperature-controlled room at 25°C under a 12-h light/dark cycle. Male diabetic KKAy/Ta Jcl (KKAy) mice and male C57BL/6 mice were purchased from Nippon CLEA (Tokyo, Japan). We utilized C57BL/6 mice as a control consistent with several other reports [19], [20]. The KKAy and C57BL/6 mice were divided into 2 groups, which were administered NO-NIF or vehicle, respectively. NO-NIF (30 mg/kg) suspended in normal saline containing 1% carboxymethylcellulose (CMC) was injected intraperitoneally for 4 weeks (at 12–16 weeks of age). Vehicle (1% CMC in normal saline) was also injected intraperitoneally at the same volume as NO-NIF for 4 weeks. The eNOS knockout mice (B6.129P2-*Nos3^tm1Unc^*/J) were purchased from the Jackson Laboratory (Bar Harbor, ME, USA). Male eNOS knockout mice were divided into 2 groups that were administered NO-NIF (30 mg/kg) or vehicle intraperitoneally for 4 weeks (at 12–16 weeks of age).

### Systolic blood pressure (SBP)

SBP was monitored using tail-cuff plethysmography (BP-98A; Softron Co., Tokyo, Japan). A set of 10 measurements was averaged for each animal.

### Blood chemistry

Blood glucose levels were monitored using a G checker kit (Sanko Junyaku CO., Ltd., Tokyo, Japan). Serum was collected during animal dissection. The serum insulin concentration was measured using Morinaga ultrasensitive mouse insulin assay kit (Morinaga Institute of Biological Science, Ltd., Tokyo, Japan). Serum creatinine concentration was measured using Creatinine-test-Wako kit (Wako Pure Chemical Industries, Ltd., Osaka, Japan). Blood urea nitrogen (BUN) was determined using BUN-test-Wako kit (Wako Pure Chemical Industries, Ltd.).

### Urinary measurements

Urine samples were collected for 24 h by using a metabolic cage (Nippon CLEA). Urinary creatinine and *N*-acetyl-β-d-glucosaminidase (NAG) levels were determined using an enzymatic method (SRL, Tokyo, Japan). The urinary albumin concentration was measured with a mouse albumin enzyme-linked immunosorbent assay (ELISA) kit (Shibayagi Co., Ltd., Shibukawa, Japan). The urinary total protein concentration was quantified with the Bradford protein assay (Bio-Rad Laboratories, Hercules, CA, USA). The urinary AGT concentration was measured using mouse total AGT assay kit (Immuno-Biological Laboratories) according to the manufacturer's instructions.

### Intraperitoneal glucose tolerance test (IPGTT) and insulin tolerance test (ITT)

Blood was drawn from the tail vein at scheduled time points. When performing the IPGTT, the mice were injected with 20% glucose solution (2.0 g/kg, i.p.) after a 16 h fast, and the blood glucose level was measured prior to and 15, 30, 60, 90, 120, and 180 min after the glucose injection. The area under the blood concentration versus time curve for glucose within 120 min after administration of the glucose solution was calculated using the trapezoidal rule. The mice were injected with insulin (1.0 IU/kg, i.p.; Humulin R; Eli Lilly, Indianapolis, IN, USA) after a 4-h fast, and the blood glucose level was measured prior to and 15, 30, 60, 90, and 120 min after insulin injection.

### Tissue preparation

At the endpoint of the tests, the animals were anesthetized by injection of sodium pentobarbital (150 mg/kg, i.p.) and euthanized by cervical dislocation. The thoracic aorta, epididymal fat, and kidneys were resected and placed in 4% paraformaldehyde or snap frozen.

### Histological analysis

Paraffin-embedded kidney samples were cut into 2-μm sections and stained with periodic acid-Schiff (PAS) reagent. To analyze the mesangial expansion, the glomerular diameter and glomerular tuft area were determined from the average of 20 glomeruli randomly selected from the mice by using ImageJ 1.38 software (National Institutes of Health, Bethesda, MD, USA). White adipose tissue (10-μm thickness) was stained with hematoxylin and eosin (H&E). Adipocyte size was determined by the average of 5 different regions in each sample.

### Immunohistochemical staining

Paraffin-embedded kidney tissue samples were sectioned and deparaffinized. After antigen retrieval, the tissue samples were incubated with the primary antibody at 4°C overnight. The antibody distribution pattern was visualized using a streptavidin-biotin complex assay and DAB substrate kit (Vector Laboratories, Burlingame, CA, USA). Sections incubated without the primary antibody were used as negative controls. Hematoxylin was used for counter staining.

### ROS detection in kidney

DHE (10 μM) was used to evaluate the *in situ* production of superoxide in cryosections of the kidney as previously described [17].

### Systemic oxidative stress markers

The levels of urinary 8-hydroxy-2′-deoxyguanosine (8-OHdG) were determined using an ELISA kit (New 8-OHdG Check, Japan Institute for the Control of Aging, Nikken SEIL Co., Shizuoka, Japan). Urinary 8-isoprostane excretion was measured using an 8-isoprostane EIA Kit (Cayman Chemical, Ann Arbor, MI, USA).

### Superoxide dismutase activity

Superoxide dismutase (SOD) activity was measured by competitive inhibition assay using a SOD assay kit-WST (Dojindo, Kumamoto, Japan) according to the manufacturer's instructions. Whole kidney tissues were homogenized and the total protein concentration was determined by Bradford protein assay. Enzymatic activity was expressed in units per mg protein.

### Measurement of mitochondrial ROS

Mitochondrial ROS production in HGECs was measured using MitoSOX, a fluorescent probe specific for mitochondrial superoxide (Life Technologies, Carlsbad, CA, USA). MitoSOX (5 μM) was added to the medium and incubated for 30 min at 37°C. Cellular fluorescence was examined under a fluorescence microscope at excitation and emission of 510 and 580 nm, respectively.

### Western blotting

Western blotting for protein analysis was performed as described previously [21] by using antibodies to ICAM-1 (1∶1000) and β-actin (1∶1000) (Cell Signaling Technology, Inc., Beverly, MA, USA).

### Quantitative real-time PCR

The procedures for total RNA extraction from the kidney, cDNA synthesis, and quantitative real-time PCR have been previously described [22]. The primers used were as follows: 5′-TCACCAGGAATGTGTACCTGAC-3′ and 5′-GGCTTGTCCCTTGAGTTTTATGG-3′ for ICAM-1, and 5′-AAGTGTGACGTTGACATCCG-3′ and 5′-GATCCACATCTGCTGGAAG-3′ for β-actin.

### Statistical analysis

The values for each parameter within a single group are expressed as the means ± SEM. One-way analysis of variance was used to determine the statistical significance among groups, after which a modified t-test with Bonferroni correction was used for comparison between groups. Values of p<0.05 were accepted as statistically significant.

## Results

### NO-NIF had no effect on glucose metabolism

**Figure 1:**
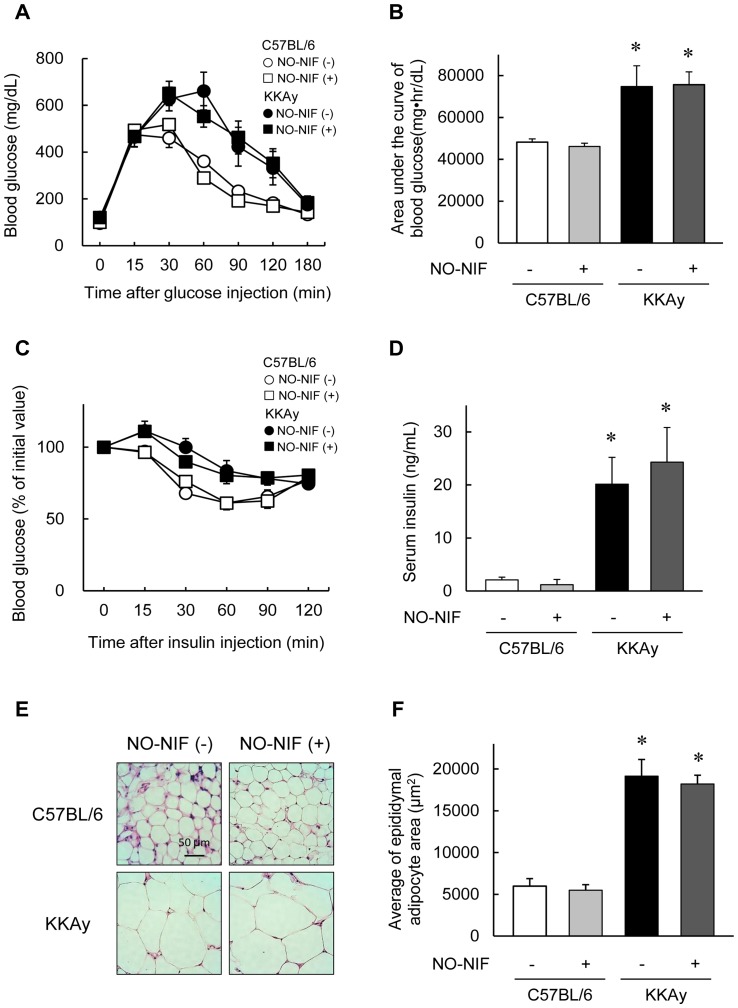
NO-NIF had no effect on glucose metabolism. (A) Changes in the blood glucose level during IPGTT. (B) The area under the blood concentration versus time curve for glucose during IPGTT. Values are expressed as the means ± S.E., n = 8–10. *p<0.05 vs. vehicle-treated C57BL/6 mice. (C) Changes in the blood glucose level during ITT. Values are expressed as a percentage of the initial value. (D) Levels of serum insulin. Values are expressed as the means ± S.E., n = 8–10. *p<0.05 vs. vehicle-treated C57BL/6 mice. (E) Representative images showing hematoxylin-eosin staining of adipocytes in epididymal fat of the C57BL/6 and KKAy mice with or without NO-NIF. (F) Average of the epididymal adipocyte size. Values are expressed as the means ± S.E., n = 8–10. *p<0.05 vs. vehicle-treated C57BL/6 mice.

Compared to age-matched C57BL/6 mice, 16-week-old KKAy mice showed increased body weight, fasting plasma glucose levels, and SBP (Table 1). NO-NIF (30 mg/kg, i.p.) had no effect on these parameters in either the C57BL/6 or KKAy mice. NO-NIF also showed no influence on glucose tolerance or insulin tolerance, both of which were impaired in the KKAy mice (Figure 1A, B, and C). The serum insulin level was elevated in the KKAy mice irrespective of NO-NIF administration (Figure 1D). The KKAy mice exhibited adipocyte hypertrophy, as estimated by the white adipose tissue weight and adipocyte size, neither of which was affected by NO-NIF administration (Table 1, Figure 1E, and F).

**Table 1:** Physiological effects in C57BL/6 and KKAy mice 4 weeks after nitrosonifedipine (NO-NIF) administration.

NO-NIF	C57BL/6		KKAy	
	−	+	−	+
Body weight (g)	28.1±0.6	27.7±0.4	49.7±0.9^*^	47.6±0.6^*^
Fasting plasma glucose (mg/dL)	95.8±5.4	99.3±7.8	120.8±9.0^*^	120.0±11.8^*^
Systolic blood pressure (mmHg)	94.4±2.5	96.0±2.6	111.1±2.3^*^	108.3±3.3^*^
White adipose tissue weight (g)	0.8±0.1	0.6±0.1	1.9±0.1^*^	1.7±0.1^*^
Kidney weight (g)	360.8±9.2	374.5±11.1	645.9±20.5^*^	614.0±21.1^*^
Serum creatinine (mg/dL)	0.112±0.005	0.103±0.005	0.091±0.004	0.090±0.005
Urine volume (mL)	0.8±0.1	0.9±0.2	7.4±1.3^*^	3.7±0.5^*#^
Daily water intake (mL/day)	10.3±1.6	10.2±1.4	20.2±1.8^*^	18.2±1.4^*^

Values are expressed as mean ± SEM, n = 8–10. *p<0.05 vs. vehicle-treated C57BL/6 mice, #p<0.05 vs. vehicle-treated KKAy mice.

### NO-NIF attenuated the progression of renal injury in the KKAy diabetic mice

**Figure 2:**
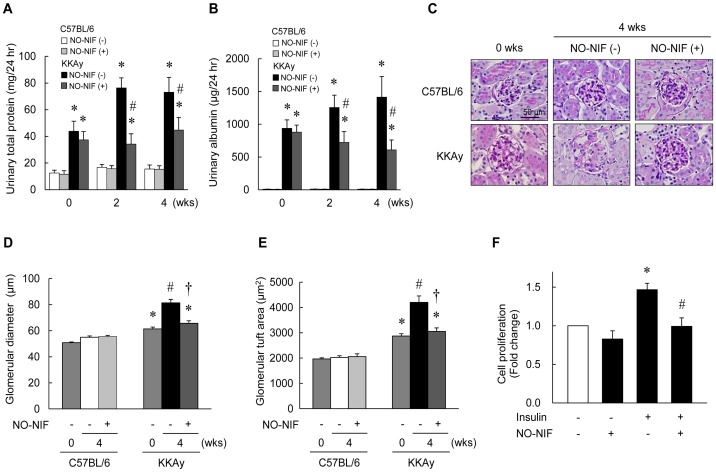
Effects of NO-NIF on DN in KKAy mice. Changes in urinary total protein excretion (A) and urinary albumin excretion (B) of the C57BL/6 and KKAy mice with or without NO-NIF at 0, 2, and 4 weeks after the commencement of NO-NIF administration. Values are expressed as the means ± S.E., n = 8–10. *p<0.05 vs. C57BL/6 mice at 0 weeks, #p<0.05 vs. vehicle-treated KKAy mice at 4 weeks. (C) Histopathological analysis of diabetic kidneys at 0 and 4 weeks after the commencement of NO-NIF administration. Representative histological images of PAS staining. Quantitative analysis of the glomerular diameter (D) and glomerular tuft area (E) in the C57BL/6 and KKAy mice. Values are expressed as the means ± S.E., n = 8–10. *p<0.05 vs. C57BL/6 mice at 0 weeks, #p<0.05 vs. vehicle-treated C57BL/6 mice at 4 weeks, and †p<0.05 vs. vehicle-treated KKAy mice at 4 weeks. (F) The effect of NO-NIF on insulin-induced HMC proliferation. HMCs were treated with 10 μM NO-NIF for 6 h prior to treatment with 100 nM insulin for 48 h. Cell proliferation was determined using the MTT assay according to the manufacturer's instructions. *p<0.05 vs. control, #p<0.05 vs. insulin alone.

As shown in Table 1, kidney weight, creatinine clearance, and urine volume were significantly increased in the 16-week-old KKAy mice compared to those in the C57BL/6 mice. However, NO-NIF administration had no effect on kidney weight or creatinine clearance in either mouse strain. The KKAy mice exhibited a significant exacerbation of urinary albumin and urinary total protein excretion compared to that observed in the C57BL/6 mice at 12 to 16 weeks of age (Figure 2A and B). However, NO-NIF administration inhibited this exacerbation in the KKAy mice (Figure 2A and B). Histological test revealed glomerular expansion as estimated by the glomerular diameter and glomerular tuft area in the KKAy mice. However, NO-NIF administration prevented further expansion of the glomeruli (Figure 2C–E). The increase in the glomerular tuft area indicates an increase in the mesangial matrix and mesangial cell (MC) proliferation. NO-NIF significantly inhibited insulin-induced cultured human MC proliferation (Figure 2F).

### NO-NIF inhibited endothelial damage and renal tubular injury in animal models of diabetes mellitus

**Figure 3:**
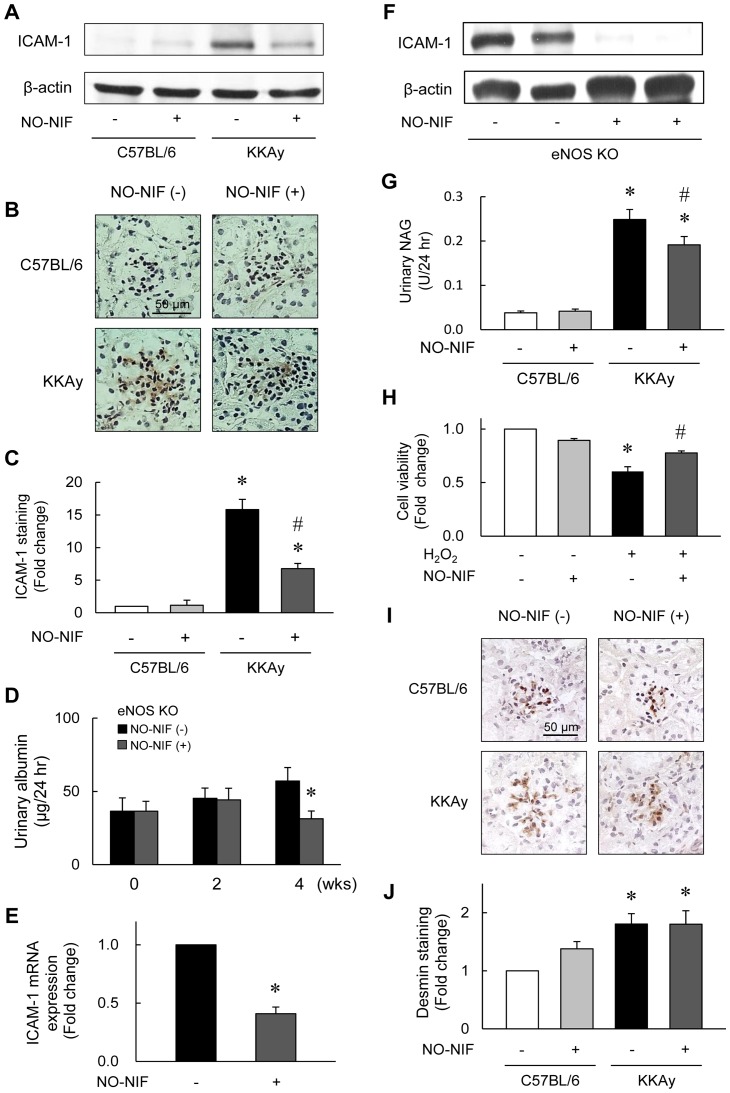
NO-NIF improved endothelial dysfunction and renal tubular injury in DN. Immunoblotting (A) and immunohistochemistry (B and C) for ICAM-1 expression in the diabetic kidney of the C57BL/6 and KKAy mice with or without NO-NIF 4 weeks after the commencement of NO-NIF (30 mg/kg) administration. (A) Representative blot of ICAM-1 and β-actin. Equal amounts of protein in each sample were separated by SDS-PAGE and analysis for ICAM-1 by western blotting. (B) Representative immunohistochemical staining of ICAM-1 in glomeruli. (C) Quantitative analysis for staining of ICAM-1 in glomeruli. Values are expressed as the means ± S.E., n = 8–10. *p<0.05 vs. vehicle-treated C57BL/6 mice, #p<0.05 vs. vehicle-treated KKAy mice. (D) Changes in the urinary albumin excretion of eNOS knockout mice with or without NO-NIF (30 mg/kg) at 0, 2, and 4 weeks after the commencement of NO-NIF administration. Values are expressed as the means ± S.E., n = 8. *p<0.05 vs. vehicle-treated KKAy mice at 4 weeks. (E) Quantitative analysis for mRNA expression of ICAM-1. The cDNA was synthesized from the thoracic aorta tissues of eNOS knockout mice, and quantitative real-time PCR was performed using primers for ICAM-1. The mRNA expression level was normalized to that of the β-actin gene. (F) Representative blot of ICAM-1 and β-actin in the thoracic aorta of eNOS knockout mice. (G) Changes in the urinary NAG excretion in the C57BL/6 and KKAy mice with or without NO-NIF treatment (30 mg/kg) 4 weeks after the commencement of NO-NIF administration. Values are expressed as the means ± S.E., n = 8–10. *p<0.05 vs. vehicle-treated C57BL/6 mice, #p<0.05 vs. vehicle-treated KKAy mice. (H) HK-2 cells were preincubated with 10 μM of NO-NIF for 6 h and then exposed to 100 μM H_2_O_2_ for 24 h. The cell viability was assessed using an MTT assay according to the manufacturer's instructions. *p<0.05 vs. control, #p<0.05 vs. H_2_O_2_ alone. (I) Representative immunohistochemical staining of desmin in glomeruli. (J) Quantitative analysis for the staining of desmin in glomeruli. Values are expressed as the means ± S.E., n = 8–10. *p<0.05 vs. vehicle-treated C57BL/6 mice.

The protein expression of ICAM-1, which is a marker of EC injury in the glomerulus, was significantly higher in the kidneys of the KKAy mice compared to that in the kidneys of the C57BL/6 mice; this difference was reduced by NO-NIF administration to the KKAy mice (Figure 3A–C). To further elucidate the effect of NO-NIF against EC damage, NO-NIF was administered to eNOS knockout mice. The increased urinary albumin and the expression of ICAM-1 in the aorta of the eNOS knockout mice were significantly suppressed by NO-NIF administration (Figure 3D–F). These results suggest that NO-NIF protects the kidneys against EC damage, even in the absence of eNOS.

The excretion of NAG in urine, an indicator of renal tubular dysfunction, was also significantly higher in the KKAy mice compared to that in the C57BL/6 mice, and NO-NIF significantly attenuated these levels in the KKAy mice (Figure 3G). As shown in Figure 3H, the reduction in cell viability induced by H_2_O_2_ stimulation was blocked by NO-NIF pretreatment in HK-2 cells, an immortalized proximal tubule epithelial cell line from normal adult human kidney. These results suggest that NO-NIF exerts a protective effect against tubular epithelial as well as EC damage. On the other hand, NO-NIF had no effect on the increased protein expression of desmin, a marker of podocyte injury, in the KKAy mice (Figure 3I and J).

### NO-NIF suppressed oxidative stress in the KKAy mice and in animal models of endothelial injury

**Figure 4:**
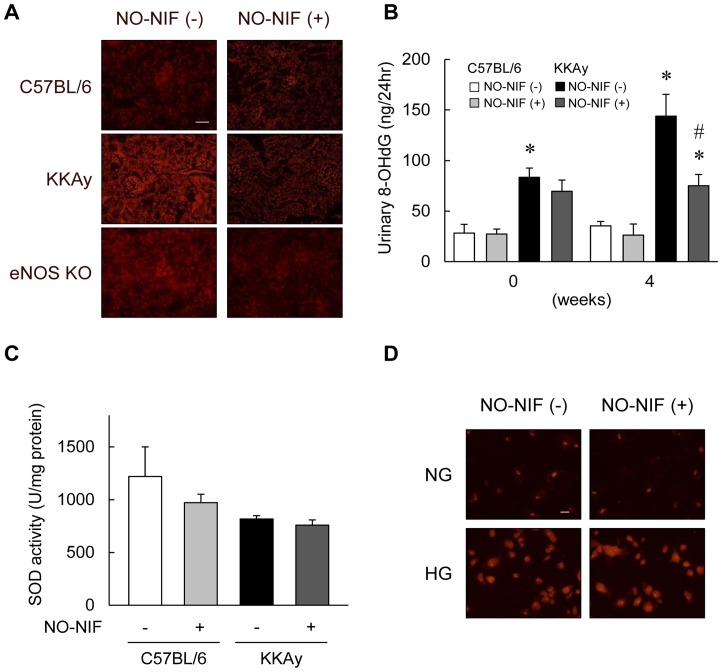
NO-NIF suppressed oxidative stress in a diabetic and endothelial injury model. (A) Kidney superoxide production was evaluated by DHE staining. Representative images of DHE stained sections from the kidney of the C57BL/6, KKAy and eNOS knockout mice. Scale bar; 100 μm. (B) Changes in urinary 8-OHdG of the C57BL/6 and KKAy mice with or without NO-NIF at 0 and 4 weeks after the commencement of NO-NIF administration. The levels of urinary 8-OHdG were measured by enzyme-linked immunosorbent assay. Values are expressed as the means ± S.E., n = 8–10. *p<0.05 vs. vehicle-treated C57BL/6 mice, #p<0.05 vs. vehicle-treated KKAy mice. (C) Change in SOD activity in the kidney of the C57BL/6 and KKAy mice with or without NO-NIF 4 weeks after the commencement of NO-NIF administration. SOD activity was measured by competitive inhibition assay using a SOD assay kit-WST. Values are expressed as the means ± S.E., n = 8–10. (D) The effect of NO-NIF on HG-induced mitochondrial ROS production using MitoSOX red in HGECs. HGECs were preincubated with 10 μM of NO-NIF for 6 h, and then exposed to 30 mM HG for 18 h. Representative images are shown. Scale bar, 100 μm.

As shown in Figure 4A, DHE fluorescence intensity, indicating superoxide generation, was enhanced in the kidneys of the KKAy mice and eNOS knockout mice as compared to that in the kidneys of the C57BL/6 mice. However, NO-NIF administration suppressed this enhanced DHE fluorescence intensity in the KKAy and eNOS knockout mice (Figure 4A). The urinary level of 8-OHdG, a systemic oxidative stress marker, was elevated in the KKAy mice compared to that in the C57BL/6 mice, and was decreased by NO-NIF administration (Figure 4B). Levels of urinary 8-isoprostane, a lipid peroxidation marker, were significantly increased in l-NAME-treated rats and reduced by NO-NIF administration (Supplementary Results in File S1: Effect of NO-NIF on urinary 8-isoprostane in l-NAME treated rats). On the other hand, there was no difference in the SOD activity in the kidney between the KKAy and C57BL/6 mice with or without NO-NIF administration (Figure 4C). Because high glucose (HG)-induced oxidative stress is mainly due to mitochondrial superoxide [2], [23], [24], the effect of NO-NIF on HG-induced ROS production was visualized using MitoSOX red in HGECs. MitoSOX fluorescence was enhanced by HG stimulation, and NO-NIF treatment did not significantly suppress this mitochondrial superoxide generation (Figure 4D).

### NO-NIF inhibited intrarenal AGT expression

**Figure 5:**
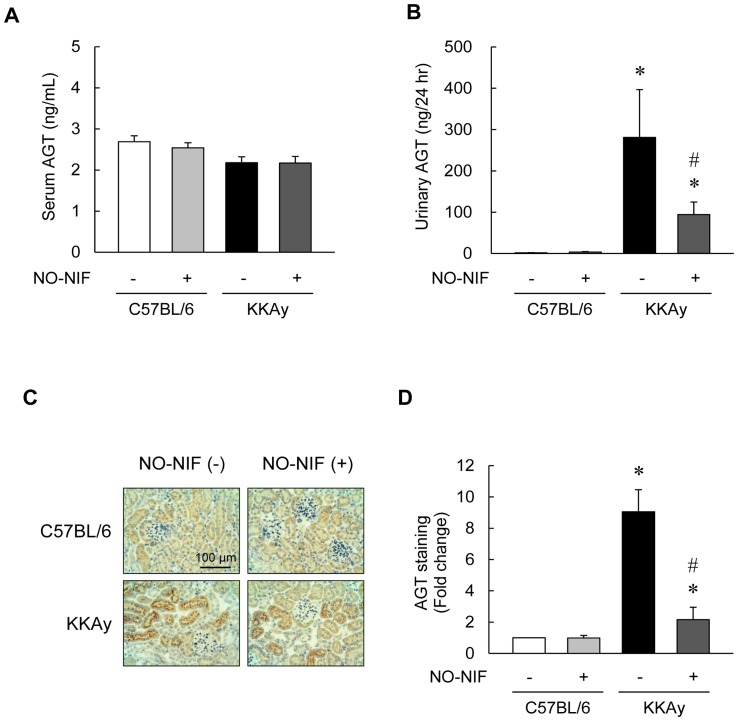
NO-NIF inhibited intrarenal AGT expression in KKAy mice. (A) The concentrations of serum AGT in the C57BL/6 and KKAy mice with or without NO-NIF at 4 weeks after the commencement of NO-NIF administration were measured with a mouse total AGT ELISA kit. (B) Changes in the urinary AGT excretion in the C57BL/6 and KKAy mice with or without NO-NIF at 4 weeks after the commencement of NO-NIF administration. Values are expressed as the means ± S.E., n = 8–10. *p<0.05 vs. vehicle-treated C57BL/6 mice at 4 weeks, #p<0.05 vs. vehicle-treated KKAy mice at 4 weeks. (C) Representative immunohistochemical staining of AGT in kidney. Proximal tubular AGT immunostaining was more intense in the KKAy mice than in the C57BL/6 mice. (D) Quantitative analysis of the staining of AGT in kidney. The fraction of immuno-reactive area (brown) was measured using the EIS-Elements software (Nikon Corporation, Tokyo, Japan). At least 20 consecutive microscopic fields were examined for each slide. Values are expressed as the means ± S.E., n = 6. *p<0.05 vs. vehicle-treated C57BL/6 mice, #p<0.05 vs. vehicle-treated KKAy mice.

The increase in AGT in the kidney or urine has been reported to correlate with intrarenal RAS activation and subsequent ROS generation [6], [8], [9], [25]. Moreover, intrarenal AGT is increased in diabetic patients and in rat models of diabetes [6], [8], [9], [25]. Although we found no significant differences in the serum AGT levels between the KKAy and C57BL/6 mice with or without NO-NIF (Figure 5A), urinary AGT was markedly increased in the KKAy mice compared to that in the C57BL/6 mice. This difference was reduced by NO-NIF treatment in the KKAy mice (Figure 5B). As shown in Figure 5C and D, immunohistochemical staining revealed that renal AGT was predominantly localized in proximal tubular cells in the KKAy mice, and this expression was significantly suppressed by NO-NIF administration. These results imply that NO-NIF suppresses intrarenal RAS activation in DN, and further suggest that the antioxidative effect of NO-NIF on DN is mediated through intrarenal AGT.

## Discussion

The results from the present study indicate that the antioxidant activity of NO-NIF is effective against type 2 DN that is accompanied by an increase in both intrarenal AGT and EC injury. Our results also shed light on the antioxidative mechanisms of NO-NIF as well as highlight its potential as a novel therapeutic candidate in DN.

The development and progression of DN is highly complex, given the diversity of the cell populations present within the kidney and the various physiological roles played by this organ. It is well known that DN presents with abnormal findings in various cell types resident in kidney, including ECs, MCs, podocytes, and proximal tubular cells [3], [26], [27]. In previous reports we showed that NO-NIF reduced Ang II-induced vascular remodeling by ameliorating the damage to vascular smooth muscle cells and ECs [17]. We also showed that NO-NIF improved the vascular endothelial dysfunction induced by l-NAME treatment in rats [18]. Results from others have also suggested that NO-NIF prevented ECs from oxidative stress-induced cytotoxicity in an *in vitro* study [16]. In accord with these previously reported findings, NO-NIF suppressed endothelial damage in the kidneys of the KKAy mice (Figure 3A–C). Interestingly, NO-NIF administration suppressed EC injury and improved renal disease even in eNOS knockout mice, which were used as a model of vascular endothelial dysfunction-induced renal failure (Figure 3D–F). These findings suggest that NO-NIF protects the kidney against EC damage independently of eNOS.

In addition to the beneficial effects on renal ECs, NO-NIF also exhibited a protective effect against damage to MCs (Figure 2F) and renal tubular cells (Figure 3G and H) *in vivo* and *in vitro*. On the other hand, NO-NIF had no effect on damaged podocytes (Figure 3I and J) or on the abnormality of adipocytes (Figure 1E and F) in the KKAy mice. These results imply that the effects of NO-NIF are specific to certain cells or tissues. Such specificity was also implied by the finding that NO-NIF had no effect on glucose metabolism in the KKAy mice, that is, NO-NIF administration did not lower the fasting blood glucose level (Table 1), nor did it effect the impaired glucose or insulin tolerance in the KKAy mice (Figure 1A–D). Thus, NO-NIF appears specifically effective against renal disease in type 2 diabetes. However, our results also clearly indicate that the improvement in DN by NO-NIF was independent of a blood glucose lowering effect.

We previously reported that NO-NIF is converted to its radical form when incubated with unsaturated fatty acids, which are major components of cell membranes or other lipid bilayers, or with cultured human umbilical vein ECs (HUVECs) [15]. NO-NIF potently scavenges DPPH radicals when reacted with unsaturated fatty acids [15]. We further demonstrated that NO-NIF treatment altered cell membrane fluidity in cultured vascular smooth muscle cells (VSMCs) and that NO-NIF can scavenge the NADPH oxidase-derived ROS induced by Ang II [17]. Together, these findings indicate that NO-NIF functions as an antioxidant at the cell membrane.

As shown in Figure 4A, the DHE fluorescence intensity indicating ROS generation was suppressed by NO-NIF administration in the kidneys of eNOS knockout mice. In l-NAME-administered rats, the levels of urinary 8-isoprostane, a systemic lipid oxidation marker, were significantly reduced by NO-NIF administration (Figure S1). It has been reported that superoxide production is increased in the absence of NO in eNOS knockout mice and l-NAME-administered rats [28], [29]. We have previously reported that NO-NIF attenuates the superoxide-derived free radicals in HUVECs [15]. Based on the results obtained using a model of endothelial damage, NO-NIF significantly inhibited the EC damage-related oxidative stress induced by NO deficiency via its antioxidative action. In the KKAy mice, NO-NIF significantly suppressed systemic and renal ROS generation. However, SOD activity was unchanged by NO-NIF administration (Figure 4C), supporting the notion that NO-NIF exerts an effect on the ROS generative pathway and not the reducing pathway.

Because numerous studies have shown that HG-induced oxidative stress causes mitochondrial abnormalities, including excess superoxide generation [2], [23], [24], we tested the effect of NO-NIF on HG-induced mitochondrial ROS *in vitro*. NO-NIF treatment did not significantly attenuate the mitochondrial ROS generation induced by HG (Figure 4D) in HGECs. Correspondently, we confirmed that NO-NIF had no effect on HG-induced ICAM-1 expression in HGECs (Figure S2 A and B). From these results, NO-NIF should have no direct effect on the HG-induced signaling pathway in HGECs. Nifedipine is lipophilic and preferentially accumulates in the cytoplasmic membrane [30]. NO-NIF has a similar chemical structure and also accumulates in cytoplasmic membranes [11]. It has been hypothesized that NO-NIF cannot reach the mitochondrial membrane by traveling through the cytosol because of its lipophilicity [11], [15]. We have reported previously that NO-NIF reacts directly with unsaturated fatty acids [15], and that NO-NIF reacts with the cell membrane to alter membrane fluidity [17]. Together these findings suggest that the antioxidative action of NO-NIF is exerted at or near the cytoplasmic membrane, not in the cytoplasm, which includes mitochondria. It is implied that NO-NIF would not exert a beneficial influence on renal injury caused by hyperglycemia alone because of both its chemical properties and its site of action. In addition, type 2 DN is caused by many other factors such as hyperinsulinemia, inflammatory cytokines, and hyperglycemia as well as by an enhancement in the RAS [1], [2], [3], [6]. In the present study, we showed NO-NIF significantly inhibited insulin-induced MC proliferation (Figure 2F) and suppressed the reduction of cell viability induced by H_2_O_2_ in HK-2 cells (Figure 3H). We previously reported that NO-NIF significantly inhibited the cytotoxicity of TNF-α in HGECs [16] and suppressed Ang II-induced ROS in VSMCs [17]. Hence, we propose that NO-NIF has multifaceted effects and plays a unique role as a new type of antioxidant that possesses a plasma membrane protective effect and is effective against type 2 DN.

At present, the only available strategy for treating DN is the use of RAS blocking drugs, such as angiotensin converting enzyme inhibitors or Ang II type 1 receptor blockers (ARB) [31], [32], [33]. An activator of Nrf2 (which is a transcriptional factor regulating oxidative stress and inflammation) bardoxolone was developed as a novel anti-DN agent; however, bardoxolone clinical trials were discontinued because of the occurrence of serious adverse events, including death [34], [35]. Vitamin E, a representative antioxidant, was reported in a large clinical trial to neither decrease the onset of cardiovascular diseases nor prevent the development of DN [36]. It is well known that intrarenal RAS induces oxidative stress and plays an important role in the progression of DN [27]. Ogawa *et al*. demonstrated that ARB reduced urinary AGT excretion as well as the levels of urinary markers of oxidative stress and inflammation in patients with type 2 DN [6]. A crystal analysis demonstrated that oxidative stress induced a conformational change in AGT to a form that more effectively releases angiotensin at the cellular level, leading to RAS activation [37]. In the present study, we examined the effects of NO-NIF on intrarenal AGT in KKAy mice and found that NO-NIF significantly inhibited AGT levels in kidney and urine (Figure 5B–D). This inhibition of intrarenal AGT correlates with the suppression of proteinuria and ROS in the kidney (Figure 2A, 4A and B). We suggest that the inhibition of intrarenal AGT by NO-NIF in KKAy mice is due to a decrease in ROS induced by several cytotoxic factors such as insulin, TNF-α, and Ang II. Although it remains unclear whether NO-NIF suppresses intrarenal AGT directly, together these findings imply that the antioxidative effects of NO-NIF are due at least in part to its inhibition of intrarenal AGT, and this might interrupt the vicious cycle of ROS-AGT-Ang II-ROS. Interestingly, even though NO-NIF suppressed intrarenal AGT, it did not affect systemic AGT (Figure 5A). Since NO-NIF does not block calcium channels, it has no direct effect on blood pressure, as confirmed both in this study (Table 1) and in our previous study [17]. Hence, NO-NIF has potential use in patients with DN even though it lacks an effect on hypertension.

In conclusion, we suggest that NO-NIF prevents the progression of type 2 DN associated with endothelial dysfunction through a specific antioxidative property that is quite different from other known antioxidants. Although further study – including an elucidation of NO-NIF pharmacokinetics – is obviously needed, NO-NIF holds promise as a novel and safe therapeutic strategy against DN.

## Supporting Information

File S1 (DOCX)

Figure S1 **Effect of NO-NIF on urinary 8-isoprostane in l-NAME-treated rats.** l-NAME (1 g/L) was administered in drinking water for 3 weeks at the same time as NO-NIF was administered. The daily intake of l-NAME was estimated to be 20–30 mg per rat. Urinary 8-isoprostane levels were measured by enzyme-linked immunosorbent assay. Values are expressed as the means ± S.E., n = 8–10. *p<0.05 vs. vehicle-treated control rats, #p<0.05 vs. vehicle-treated l-NAME rats.
(TIF)

Figure S2 **Effect of NO-NIF on high glucose-induced ICAM-1 expression in HGECs.** HGECs were preincubated with 10 μM of NO-NIF for 6 h and then exposed to HG (30 mM) for 24 h. (A) Representative blot of ICAM-1 and β-actin. Equal amounts of protein in each sample were separated by SDS-PAGE and analyzed for ICAM-1 by western blotting. (B) Results are expressed as the ratio between signals on the western blot corresponding to ICAM-1 and β-actin. Values are expressed as the means ± S.E., n = 4. *p<0.05 vs. control.
(TIF)
