# Supplementary material for: Nitrosonifedipine Ameliorates the Progression of Type 2 Diabetic Nephropathy by Exerting Antioxidative Effects
Source: PLoS One. 2014 Jan 28;9(1):e86335. doi: 10.1371/journal.pone.0086335 (PMC3904885; doi:10.1371/journal.pone.0086335)
Supplement: File S1 — (DOCX) [file pone.0086335.s001.docx]

**Supporting Information**

**Figure S1. Effect of NO-NIF on urinary 8-isoprostane in l-NAME-treated rats.**

l-NAME (1 g/L) was administered in drinking water for 3 weeks at the same time as NO-NIF was administered. The daily intake of l-NAME was estimated to be 20–30 mg per rat. Urinary 8-isoprostane levels were measured by enzyme-linked immunosorbent assay. Values are expressed as the means ± S.E., n = 8–10. *p < 0.05 vs. vehicle-treated control rats, #p < 0.05 vs. vehicle-treated l-NAME rats.

**Figure S2. Effect of NO-NIF on high glucose-induced ICAM-1 expression in HGECs.**

HGECs were preincubated with 10 μM of NO-NIF for 6 h and then exposed to HG (30 mM) for 24 h. (A) Representative blot of ICAM-1 and β-actin. Equal amounts of protein in each sample were separated by SDS-PAGE and analyzed for ICAM-1 by western blotting. (B) Results are expressed as the ratio between signals on the western blot corresponding to ICAM-1 and β-actin. Values are expressed as the means ± S.E., n = 4. *p < 0.05 vs. control.

**Supplementary Methods**

*Changes in urinary 8-isoprostane of L-NAME-treated rats with or without 3 weeks of NO-NIF administration*

l-NAME was purchased from Nacalai Tesque (Kyoto, Japan). Urinary 8-isoprostane excretion was measured using an 8-isoprostane EIA Kit (Cayman Chemical, Ann Arbor, MI, USA). Male Sprague-Dawley rats (7 weeks of age, weighing 240 to 280 g) were obtained from Japan SLC Inc. (Shizuoka, Japan). Rats were randomly divided into four groups. The rats in group 1 were untreated, while those in groups 2–4 were treated with l-NAME (1 g of l-NAME in a liter of drinking water). Group 2 and 4 rat were injected with NO-nifedipine (30 mg/kg, intraperitoneal) for 3 weeks (from 8 to 11 weeks of age).
