# Supplementary figures and images for: Nitrosonifedipine Ameliorates the Progression of Type 2 Diabetic Nephropathy by Exerting Antioxidative Effects
Source: PLoS One. 2014 Jan 28;9(1):e86335. doi: 10.1371/journal.pone.0086335 (PMC3904885; doi:10.1371/journal.pone.0086335)

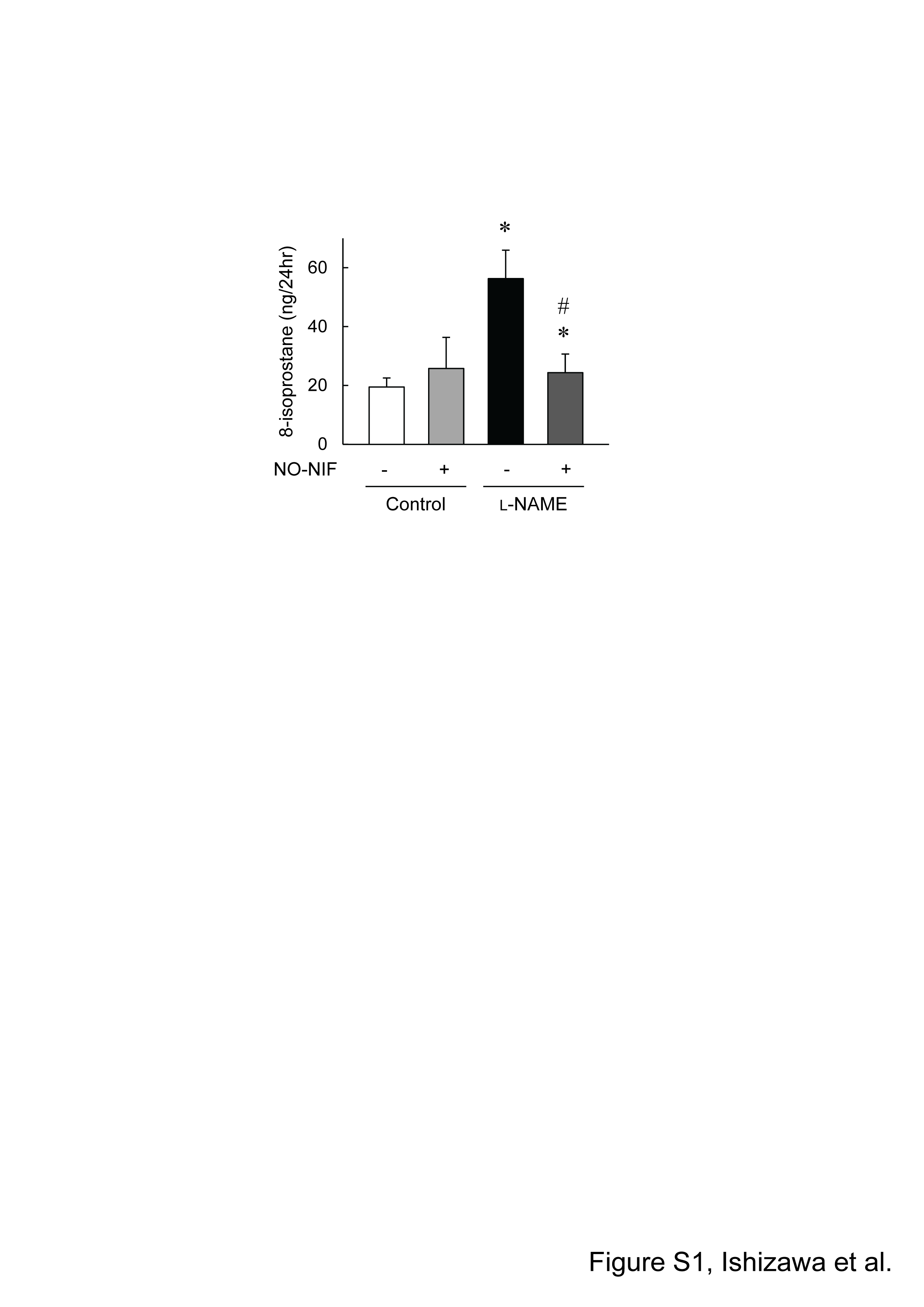

Supplement: Figure S1 — Effect of NO-NIF on urinary 8-isoprostane in l-NAME-treated rats. l-NAME (1 g/L) was administered in drinking water for 3 weeks at the same time as NO-NIF was administered. The daily intake of l-NAME was estimated to be 20–30 mg per rat. Urinary 8-isoprostane levels were measured by enzyme-linked immunosorbent assay. Values are expressed as the means ± S.E., n = 8–10. *p<0.05 vs. vehicle-treated control rats, #p<0.05 vs. vehicle-treated l-NAME rats. (TIF) [file pone.0086335.s002.tif]

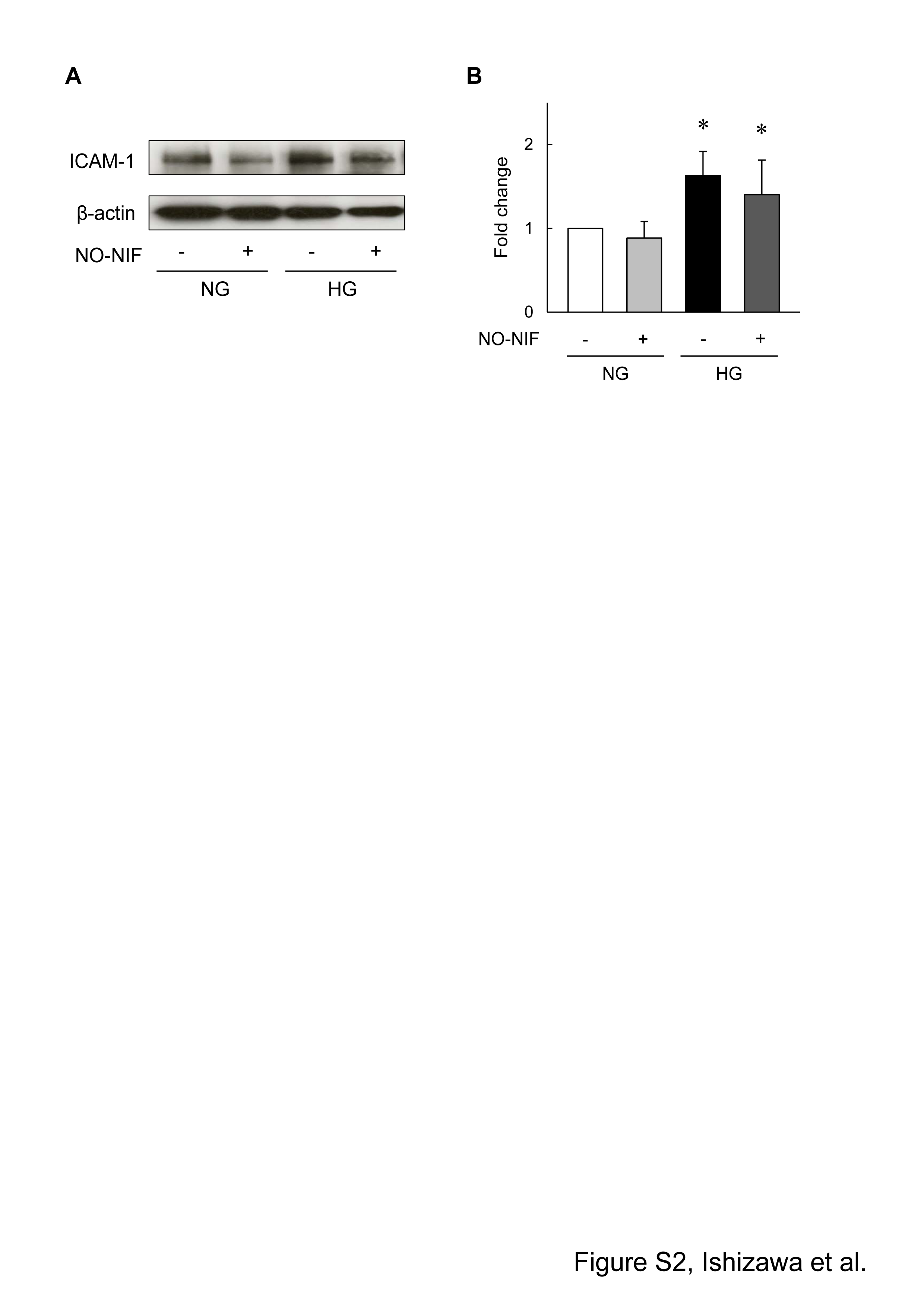

Supplement: Figure S2 — Effect of NO-NIF on high glucose-induced ICAM-1 expression in HGECs. HGECs were preincubated with 10 μM of NO-NIF for 6 h and then exposed to HG (30 mM) for 24 h. (A) Representative blot of ICAM-1 and β-actin. Equal amounts of protein in each sample were separated by SDS-PAGE and analyzed for ICAM-1 by western blotting. (B) Results are expressed as the ratio between signals on the western blot corresponding to ICAM-1 and β-actin. Values are expressed as the means ± S.E., n = 4. *p<0.05 vs. control. (TIF) [file pone.0086335.s003.tif]
